# The Early Methionine Supplementation of Ewe Lambs (F0) Modifies Meat Quality Traits of the Progeny (F1, Male Fattening Lambs)

**DOI:** 10.3390/ani15091290

**Published:** 2025-04-30

**Authors:** Mahsa Dehnavi, Javier Mateo, Alba Martín, F. Javier Giráldez, Irma Caro, Lara Morán, Sonia Andrés

**Affiliations:** 1 Instituto de Ganadería de Montaña (CSIC-Universidad de León), Finca Marzanas s/n, Grulleros, E-24346 León, Spain; mahsa.d@csic.es (M.D.); alba.martin@csic.es (A.M.); laramoran84@hotmail.com (L.M.);; 2 Departamento de Higiene y Tecnología de los Alimentos, Facultad de Veterinaria, Universidad de León, Campus Vegazana s/n, E-24071 León, Spain; jmato@unileon.es; 3 Departamento de Pediatría, Inmunología, Obstetricia y Ginecología, Nutrición y Bromatología, Psiquiatría e Historia de la Ciencia, Facultad de Medicina, Universidad de Valladolid, E-47005 Valladolid, Spain

**Keywords:** nutritional programming, postnatal oogenesis, methionine, feed efficiency, tenderness

## Abstract

Adequate management of replacement ewe lambs (F0) in dairy sheep farms during postnatal life may modify the germline cells, thus promoting transmission of intergenerational effects to the offspring (F1). To test this hypothesis, 18 newborn male lambs (F1), either born from methionine-supplemented ewe lambs (F0 ewe lambs being fed ad libitum with a milk replacer supplemented with 1 g methionine/kg DM) or not supplemented (F0 ewe lambs being fed ad libitum with the same milk replacer with no methionine added), were included in the present study. All the male F1 lambs were managed exactly in the same way along the whole lifespan in order to bring out the differences caused by methionine supplementation of F0 dams. Our data show that the methionine supplementation of dams (F0) during the suckling period did not promote significant (*p* > 0.05) changes on feed intake, growth rate, or feed efficiency of F1 male lambs during the fattening period. Moreover, the meat chemical composition (proximal, fatty acid profile, and volatile compounds) was similar for both groups (*p* > 0.05), but the meat of F1-MET lambs presented higher redness and hardness (*p* < 0.05) when compared to F1-CTRL lambs. The biochemical profile also highlighted significant (*p* < 0.05) differences in the serum creatinine and calcium content that may be at least partially related to the meat quality traits observed. Overall, all these results suggest that methionine supplementation of lambs (F0) during early postnatal life causes permanent changes in the offspring. This has positive effects, such as achieving a more attractive color of lamb meat (F1) for consumers, and negative effects, such as reduced meat tenderness.

## Simple Summary

Early feeding strategies of replacement ewe lambs (F0) may promote changes in the germ cells of the gonads from which the gametes will be derived, and, therefore, could cause intergenerational transmission of some traits to the offspring (F1). Accordingly, methionine included in milk replacers supplied to replacement ewe lambs (F0) may benefit both feed efficiency and meat quality of the male offspring (F1). The results demonstrate that feed efficiency of male F1 lambs is not modified by methionine supplied to the dams during the suckling period (F0), but redness as well as hardness of the meat are increased. These results raise concerns about the convenience of supplying methionine during the early life to ewe lambs (F0) kept in the herd for breeding and replacement purposes.

## 1. Introduction

Postnatal nutrition during the early life of ewe lambs (F0) can modify DNA methylation and therefore postnatal oogenesis during this critical window period for the neonate. Consequently, intergenerational transmission of epigenetic marks from the dams (F0) to the progeny (F1) can take place, with consequences on gene expression and phenotypic traits related to both feed efficiency and meat quality of the offspring [1,2]. Accordingly, the supply of methyl group donors [e.g., precursors of S-adenosylmethionine (SAM), the universal methyl donor involved in the methylation of all biological molecules, including DNA] has attracted attention due to their potential to induce persistent changes in both F0 and F1 [3].

Specifically, methionine (MET) serves as a crucial methyl donor in physiological intracellular processes and is also one of the most limiting amino acids required for muscle growth in ruminants [4,5]. Therefore, several studies have supplied rumen-protected methionine (0.09–0.1% diet DM) to pregnant ewes [6] and cows [7,8,9], given the fact that maternal undernutrition counteracts fetal skeletal muscle maturation during late gestation. The rationale behind this approach was trying to positively impact the number of myofibers of the fetus and their sensibility to insulin in order to improve postnatal growth rate, fatty acid metabolism, tenderness, and oxidative stability of the meat produced [10]. In this sense, Rosa-Velázquez et al. [6] observed an improvement of energy metabolism when methionine was supplied during late gestation (F0), with a concomitant enhancement of the growth observed in F1 lambs. The growth improvement of calves was also evident not only in utero but also during the postnatal life (regardless of the quality of the colostrum supplied), although the underlying mechanisms are not clear [7].

Therefore, methionine supplied during late gestation may benefit the offspring by fetal programming events. On the contrary, limited research has been conducted to clarify the impact of post-natal methionine supplementation of ewe lambs (F0) on their offspring (F1). This strategy would allow to avoid the ruminal degradation of methyl donors when supplied during mid- and late gestation. Additionally, the germline of F0 ewe lambs during this early postnatal life (e.g., suckling period) might be programmed by dietary methionine supplementation, thus promoting intergenerational transmission of traits to the F1 offspring [2]. If positive results were probed, then this post-natal approach would be especially interesting for rearing ewe lambs using milk replacers fortified with these water-soluble supplements. It must be also considered that lambs are usually reared using milk replacers formulated with cow milk powder, with a lower amount of methionine in the amino acid profile when compared to sheep milk [9]; therefore, additional benefits might be expected.

Therefore, the present study was designed to test the hypothesis that early postnatal methionine supplementation of ewe lambs (F0) induces a nutritional programming effect on their offspring, thus modifying feed efficiency and meat quality traits of the F1 male fattening lambs.

## 2. Materials and Methods

### 2.1. ARRIVE Guidelines

The study reported in the manuscript followed the recommendations in the ARRIVE guidelines. Therefore, the trial was designed with the minimum number of animals (F1, male lambs) required to have a statistical power of 80% and an expected effect size of 15%. The experimental sample size was estimated according to the standard deviation (SD) observed in previous studies of our group [11,12] for parameters such as dry matter intake, feed-to-gain ratio, cold carcass weight, dressing, proportion of first category carcass cuts, blood pCO_2_, serum concentration of urea, glucose, molar proportion of ruminal acetate, or the proportion of saturated fatty acids in meat.

### 2.2. Animals and Experimental Groups

All the details of the Assaf dairy ewes [F0, experimental flock of the Instituto de Ganadería de Montaña (CSIC, León)] giving birth to the lambs of the present study (F1, males lambs) were explained in full in Dehnavi et al. [13]. Briefly, a control group of newborn ewe Assaf lambs (F0-CTRL, *n* = 17) was fed ad libitum with a commercial milk replacer (Cordevit Calostrado, Leches Maternizadas S.A., León, Spain), whereas the second group (F0-MET, *n* = 17) received the same milk replacer supplemented with 0.1% DL-methionine (Rhodimet^®^ NP 99, Addiseo, Commentry, France) on a DM basis. After weaning (approximately 45 days of age), all F0 ewe lambs were housed together and reared under identical conditions, being artificially inseminated when they were 9 months old to produce the progeny (F1); then, the F1 lambs were assigned to two different groups (F1-CTRL and F1-MET) according to the dietary treatment received by the dams (F0). All the male F1 lambs were managed exactly in the same way along their whole lifespan to bring out the differences caused by methionine supplementation of F0 dams.

Briefly, all the F1 male newborn Assaf lambs were weighed and kept two full days with the mothers to allow colostrum intake. Thereafter, an automatic feeder was used to feed all the lambs with the same commercial milk replacer (MR, Cordevit Calostrado, Leches Maternizadas S.A., León, Spain) ad libitum until weaning, as described in detail by Giráldez et al. [14]. After approximately 6 weeks, only 18 healthy male lambs (F1-CTRL, *n* = 9 and F1-MET, *n* = 9) were weaned according to Giráldez et al. [14] and then fattened in single feedlot (floor covered by a sawdust bed and automatic drinkers available). All the lambs were offered ad libitum the same CPD that was formulated according the AFRC [15] recommendations for growing lambs when using concentrate-rich diets. Feed was delivered into automatic control feed intake devices (Agrolaval, S.L., Gijón, Spain) in order to measure individual feed intake by radio frequency identification (RFID) ear tags. Samples of the CPD offered were collected weekly and analyzed according to the procedures described by Dehnavi et al. [13]. Ingredients and chemical composition of the CPD administered are summarized in Table 1.

### 2.3. Animal Performance and Biochemical Profile

Body weight (BW) was recorded twice a week during the fattening period. The feed conversion rate (FCR) was calculated as the feed-to-gain ratio [dividing daily dry matter intake (DMI) per day by the average daily gain (ADG, g/d)]. Residual feed intake (RFI) was calculated as the difference between actual DMI and predicted DMI, which was estimated by multiple linear regression using ADG and mid-test metabolic body weight (MBW, as mid-test LBW^0.75^) as predictor variables [16].

All the animals were blood sampled at 08:30 a.m. at three time-points during the experiment (before weaning, then on day 21 of the fattening period, and the day before slaughter). Blood samples were collected by jugular venipuncture into tubes with no anti-coagulant (9 mL CAT serum Clot Activator, Greiner Bio-One GmbH, Kremsmünster, Austria); they were allowed to clot in a water bath at 37 °C for 30 min and then centrifuged at 3520× *g* for 16 min at 4 °C. Serum samples were stored at −80 °C until used to analyze the biochemical profile according to Dehnavi et al. [13]. All the biochemical parameters were determined using a clinical chemistry and turbidimetry analyzer Biosystems BA400 (Biosystems S.A., Barcelona, Spain).

### 2.4. Slaughter, Ruminal Fermentation Parameters, Carcass, and Non-Carcass Characteristics

All lambs were slaughtered after the fattening period, when they were 88 days old. Briefly, feed was withdrawn 2 h before slaughtering, then lambs were weighed to obtain the final body weight (BW). The animals were stunned by captive bolt pistol, immediately rendering them insensitive to pain. Then, they were slaughtered by exsanguination from the jugular vein, eviscerated, and skinned. After slaughter, the rumen was opened and the contents were poured into a bucket, mixed, and a sample of approximately 200 g was taken. This sample was filtered through two layers of cheesecloth to obtain a rumen fluid sample. The pH of the fluid obtained was immediately measured using a portable pH meter (Edge Meter, HANNA, Eibar, Spain). Subsequently, 40 mL of ruminal liquid was acidified with 1 mL of 20% sulfuric acid solution to stop the fermentation. Ruminal fermentation end-products [volatile fatty acids (VFA)] were measured by gas chromatography according to Carro and Miller [17].

Carcass traits were assessed as explained by Morán et al. [18] and Giráldez et al. [14]. In brief, the dressed carcass was weighed before (hot carcass weight, HCW) and after chilling at 4 °C for 24 h (cold carcass weight, CCW) to calculate chilling losses. Additionally, carcass yield was expressed as the percentage ratio of CCW to body weight (BW). The subcutaneous fat color was measured in duplicate on the lumbar region, 24 h post mortem. The pH of the *longissimus thoracis* muscle was recorded at the sixth rib on the right side at 0 h, 45 min, and 24 h post mortem. The left side of each carcass was divided into commercial cuts according to Colomer-Rocher et al. [19], and each cut was weighed to assess its proportion in the carcass. Moreover, *longissimus thoracis* (LT) *et lumborum* (LL) muscles from both half carcasses were dissected at 24 h post mortem and used for meat quality traits according to Figure S1 and Table S1.

### 2.5. Meat Chemical Composition and Fatty Acid Profile (Raw, Non-Aged Meat)

The LT portions were weighed, freeze-dried, and used for proximate composition analysis, which was carried out in duplicate following the methodology described by the AOAC [20]. Briefly, moisture was determined by weight difference after freeze drying, homogenization, and subsequent oven drying at 100 °C for 4 h. An amount of 1 g of dried muscle was used to determine the fat content with a Soxtec System 104043 extraction unit (Foss Tecator, Höganäs, Sweden) and 40–60 petroleum ether as solvent. The protein content was performed on half a gram of muscle, which was digested with 12 mL of sulphuric acid (Digestion System 6 1007 digester, Foss Tecator), with subsequent distillation (Kjeltec System 1002 Distilling unit, Foss Tecator) and titration with 0.1 M HCl. The ash was determined by ashing 1 g of dry sample in a muffle at 550 °C for 1 h using magnesium acetate and weighing the resulting residue.

The analysis of fatty acids was carried out in duplicate by extracting the fat from 1 g of freeze-dried LT muscle using a mixture of chloroform–methanol (1:1, *v*/*v*). Lipid aliquots (~10 mg) were then methylated using a basic (sodium methoxide) reagent. Fatty acid methyl esters (FAME) were analyzed using gas chromatography coupled to mass spectrometry using a 7890A gas chromatograph equipped with an HP 88 column (100 m × 0.25 mm × 0.20 mm film thickness) and coupled to a 5975C mass spectrometer (Agilent Technologies; Palo Alto, CA, USA) following Liu et al. [21] with modifications. Chromatograph conditions were as follows: helium as carrier gas (3 mL/min), injector and detector temperatures of 200 °C and 300 °C, respectively; two μL samples, a 30:1 flow split, an initial oven temperature at 170 °C, held for 24 min, and increased to 220 °C at 7.5 °C/min and at 230 °C at 10 °C/min with a hold time of 5 min, and a transfer line temperature of 230 °C. The detector operated in electron impact mode with ion voltages of 70 eV. Identification and quantification were performed according to Andrés et al. [22]. A mixture of standards (Supelco 37 Component FAMEMix; Sigma Aldrich Química, S.L., Madrid, Spain) was used for identification, as well as a comparison of the mass spectra of the peaks with those of a mass spectral library (Willey 275, Agilent Technologies). Quantification was initially calculated as a percentage of each fatty acid in total fatty acids. The fatty acid percentages were then translated into mg fatty acid/100 g of meat using both the amount of intramuscular fat content in the meat samples (g IMF/100 g meat) and the lipid conversion factor for lean lamb (0.916).

### 2.6. Lipid Oxidative Stability (Raw and Cooked Meat) and Volatile Compounds (Cooked Meat)

The levels of thiobarbituric acid reactive substances (TBARS) were measured in duplicate (non-aged) in raw LL during aerobic refrigerated storage at 4 °C on days 0, 3, and 7, using 2-cm slices (portions C, D and E, respectively; Figure S1) placed in a tray covered with cling film. TBARS levels were also assessed in cooked meat (LL), both before storage (slice F) and after 2 days of aerobic refrigerated storage (slice G), using 2-cm slices in each sample (Figure S1). The TBARS were determined following the method described by Nam & Ahn [23] with the only modification that the supernatant from the centrifugation was filtered through a filter using a 0.45 μm hydrophilic polytetrafluoroethylene syringe filter (Membrane Solutions, LLC, Auburn, WA, USA). A standard solution of 1,1,3,3-tetra-ethoxy propane was used to quantify the TBARS content of the samples. Moreover, changes in headspace volatile compounds of cooked meat were assessed in duplicate using the same storage conditions and slices as in TBARS according to Vieira et al. [24], and as modified by Carballo et al. [25]. Peak area units (AU) were used to quantify the detected compounds.

### 2.7. Color Stability (Non-Aged and Aged Raw Meat), Cooking Losses, and Texture (Non-Aged and Wet-Aged Meat After Cooking)

Two 4-cm muscle portions (one non-aged and the other 7-day aged, denoted as A and B, respectively, Figure S1) were cut crosswise into two-cm slices and placed on plastic trays with the freshly cut surface exposed to air. On day 0, after 1 h of blooming, color was measured in duplicate on the exposed surface of each slice. The slices were then stored at 4 °C under cling film and the color was assessed on days 3 and 7 on the same surface. The CIELAB values for lightness (L*), redness (a*), and yellowness (b*) were recorded using a Minolta CM-2002 chroma meter (SCI mode, D65 illuminant, 10° visual angle, 8 mm aperture; Konica-Minolta Sensing, Germany). Additionally, reflectance ratios at wavelengths 630/580 nm, 610/525 nm, and 572/525 nm were calculated from spectral data [26].

Finally, raw, non-aged, 3-day aged, and 7-day aged meat, in 7-cm portions (H, I and J, respectively, Figure S1) were cooked in a water bath at 70 °C for 40 min, and the cooking losses were measured by weight difference. Afterwards, five 1 cm × 1 cm × 3 cm prisms were obtained from the cooked portions with the long axis parallel to the muscle fibers to determinate the Warner–Bratzler shear force using a TA-XT2i analyzer (Godalming, Surrey, UK) operating at 50 mm/min test speed [27].

### 2.8. Statistical Analysis

All the analyses were performed using the SAS software (SAS 9.4 TS, SAS Inst. Inc., Cary, NC, USA). GLM procedure was used to perform one-way and factorial analysis of variance and MIXED procedure for repeated measurement analysis. The level of significance was determined at *p* < 0.05, and means were separated using the least significant difference procedure. For all variables, to assess data normality and homogeneity of variance, the Shapiro–Wilk and Levene’s tests were used, respectively.

Animal performance, ruminal parameters, and carcass and meat chemical composition data (including fatty acid profile) were subjected to one-way analysis, according to the following experimental model: Y_ij_ = µ + D_i_ + e_ij_, where Y_ijk_ is each individual observation, µ is the overall mean, D_i_ is the effect of diet (CTRL vs Methionine) and e_ij_ is the residual error. The animal was considered as the experimental unit.

Raw and cooked meat TBARS values, volatile compounds in cooked meat, cooking losses, and texture data were subjected to a factorial analysis of variance, using the following model: Y_ijk_ = µ + D_i_ + T_j_ + (DxT)_ij_ + e_ijk_, where Yijk is each individual observation, µ is the overall mean, D_i_ is the effect of diet, T_j_ is the effect of storage time, DxT is the effect of interaction between diet and storage time, and and eijk is the residual error. Meat slice was considered as the experimental unit.

Meat color data were analyzed by repeated measurements using the MIXED procedure. The model used was Y_ijk_ = µ + D_i_ + AG_j_ + (DxAG)_ij_ + S(DxA)_ijk_ + T_l_ + (DxT)_il_ + (AxT)_jl_ + (DxAxT)_ijl_ + eijklm, where Yijk is each individual observation for each variable, µ is the overall mean, D_i_ is the effect of diet, A_j_ is the effect of Aging, (DxA)_ij_ is the interaction between diet and aging, T_l_ is the effect of storage time, (DxT)_il_, (AxT)_jl,_ and (DxAxT)_ijl_ are the interactions of storage time, and e_ijkl_ is the residual error. As aging time was assessed using different slices of meat in each lamb, the effect of slice nested to diet and aging [S × (D × A)_ijk_] was used as error to evaluate the effects of diet, aging and the interaction. The effects of storage time and the rest of the interactions were contrasted with the residual error.

## 3. Results

### 3.1. Animal Performance and Biochemical Profile

Regarding animal performance, no significant differences (*p* > 0.05) were observed between F1-CTRL and F1-MET Assaf lambs during the suckling period. All lambs had similar birth weight and average daily gains during the suckling phase, so they were weaned with the same live body weight being 45–48 days old (Table 2). During the fattening phase, no significant differences were observed in DMI (*p* = 0.348), RFI (*p* = 0.679) or feed conversion rate (*p* = 0.451). All the animals were slaughtered at approximately 29 kg.

Regarding biochemical profile (Table 3), no significant (*p* > 0.05) differences of the dietary treatment (F1-CTRL vs. F1-MET) were observed for most of the parameters, but serum creatinine (0.526 vs. 0.567 mg/dL; *p* = 0.034) and calcium (11.24 vs. 11.58 mg/dL; *p* = 0.016) concentrations were significantly increased in the F1-MET lambs along the whole life.

### 3.2. Ruminal Fermentation Parameters, Carcass, and Non-Carcass Characteristics

In consonance with the lack of differences in the feed efficiency of F1 male lambs, neither significant (*p* > 0.05) difference was observed in ruminal pH at slaughter or volatile fatty acids (Table 4), thus suggesting the lack of effects of early methionine supplementation of ewe lambs (F0) on the ruminal fermentation parameters of the male offspring (F1 fattening lambs).

Dietary methionine supplementation in dams did not affect (*p* > 0.05) carcass weight, carcass yield, subcutaneous fat color, or the proportion of different commercial cuts in the offspring (F1). No significant differences (*p* > 0.05) were observed in any of the organs or fat depots.

### 3.3. Meat Chemical Composition and Fatty Acid Profile (Raw, Non-Aged Meat)

No significant (*p* > 0.05) differences promoted by methionine dietary supplementation of F0 ewe lambs were observed in meat composition of the F1 male fattening lambs (Table 5). Specifically, intramuscular fat, the component with the highest variability and effect on meat quality traits, was similar in both experimental groups (F1-CTRL vs. F1-MET, *p* = 0.757, Table 5). The fatty acid profile of intramuscular fat of F1 lambs was also unaffected (*p* > 0.05) by the methionine dietary supplementation of their dams.

### 3.4. Lipid Oxidation Stability (Raw and Cooked Meat) and Volatile Compounds (Cooked Meat)

The lipid oxidation stability of raw and cooked lamb meat during refrigerated storage was assessed by net TBARS formation, and the results are summarized in Table 6. As can be appreciated, TBARS was significantly increased during refrigerated storage (*p* < 0.001), particularly in the case of cooked meat. However, no differences between groups (F1-CTRL vs. F1-MET) were found (*p* > 0.05) in TBARS values, which is also in consonance with the similar amounts of intramuscular fat content (Table 5) and its almost identical fatty acid profile (Table 5).

Regarding volatile compounds, most of them originate from the thermal degradation of lipids and contribute to the flavor of the cooked meat, for which volatile aldehydes are mainly responsible [27,28]. In the present study, 31 volatile compounds were identified in the headspace of the cooked lamb meat samples analyzed on day 0 and after 2 days of refrigerated storage (Table 7). No significant differences were observed between the F1-CTRL and F1-MET groups for any of the identified compounds (*p* > 0.05), which is consistent with their similar fatty acid profile and lipid oxidation stability. Notably, after two days of aerobic refrigerated storage of the cooked meat, the concentration of several volatile compounds derived from lipid degradation increased (*p* < 0.05), namely hexanoic acid, hexanal, 2-octenal, 2-nonanal, decanal, butanol, 1–3-pentenol, 2-octenol, 2-heptanone, and 2,3-octanodione. These increments are attributed to meat oxidation and would mirror the increase in TBARS in cooked meat (Table 6).

### 3.5. Color Stability (Raw Meat), Texture, and Cooking Losses (Cooked Meat)

The results of color stability in lamb meat samples obtained from non-aged and seven-day aged LL muscle portions, which were placed in trays for a subsequent aerobic refrigeration for up to seven days, are presented in Table 8. As can be observed, a* (*p* = 0.001), b* (*p* = 0.001), and R610/R525 (*p* = 0.036) values were augmented with aging. Moreover, the storage of meat slices resulted in a significant (*p* < 0.05) increase in a* and b* values after 3 days, a decrease in a* from day 3 to 7, and a steady decrease in the R630/R580 ratio.

Regarding dietary treatment, early methionine supply to ewe lambs (F0-MET) resulted in a higher a*/b* ratio (*p* = 0.003) and R610/525 color estimator value (*p* = 0.001) in F1-MET meat (LT of the F1-MET fattening lambs), thus indicating more redness when compared to the control (F1-CTRL) group. No significant interaction (*p* > 0.05) effects between dietary treatment and aging or storage time were observed for these color parameters.

Cooking loss and texture (Warner–Bratzler shear force) of the lamb meat (LL) from the two dietary treatments were also analyzed both before and after two short periods of wet aging for 3 and 7 days. As shown in Table 9, cooking loss was not significantly affected by dietary treatment (*p* = 0.576) but increased with aging time (*p* < 0.001).

## 4. Discussion

This study investigated the hypothesis that methionine supplied to ewe lambs during the early post-natal life (suckling period of F0) would induce a nutritional programming effect on their offspring (F1), thus modifying feed efficiency and meat quality traits of the F1 male lambs (F1-MET) when compared to the control group (F1-CTRL). This effect would be mediated by differences caused in DNA methylation of the germline cells during this critical window period for the neonate of (F0), hence promoting intergenerational transmission of effects to the offspring (F1).

Regarding animal growth, feed intake and feed efficiency results suggest that early postnatal methionine supplementation of ewe lambs (F0) does not markedly influence overall performance of the male offspring (F1 fattening lambs). In line with these results, the ruminal fermentation pattern was similar in both experimental groups. In any case, it must be stated that ruminal fermentation parameters are mainly driven by the type of diet supplied to the animals [28], and in the present study all the F1 male lambs were fed exactly the same diet during the fattening period. These results agree with those presented by Rosa-Velazquez et al. [6], who suggested a sex-dependent effect on the offspring (F1) when methionine (0.1% of rumen-protected methionine) was supplied to pregnant ewes (F0); thus, a reduced BW in F1 female lambs was found, but not in F1 male lambs.

As expected, aging and storage time affect most of the meat quality parameters. As can be observed, a*, b*, and R610/R525 values were augmented with aging, probably due to changes in the muscle structure decreasing light scattering and hence increasing color intensity [29]. Likewise, the increases in a* and b* during the first 3 days of storage could be explained by an increase in oxymyoglobin concentration on the meat surface due to both weight loss and oxygen diffusion [30], and the further decrease in a* and the R630/R580 ratio by myoglobin oxidation [31]. Cooking losses and hardness were also affected by aging. Huff-Lonergan and Lonergan [32] demonstrated that this effect is promoted by postmortem proteolytic degradation of muscle proteins, which reduces both water retention capacity and shear force of the meat, the last one being inversely associated to both tenderness [33] and consumer satisfaction [34].

Although the effects of aging and storage time were similar in F1-MET and F1-CTRL lambs and no changes in animal performance or feed efficiency were observed between experimental groups, some results suggest that early post-natal methionine supplementation of F0-MET dams might have caused intergenerational transmission (via germline) of some traits to the male offspring. In this sense, the dams (F0-MET and F0-CTRL) showed differences in the methylation of genes involved in the formation of cartilaginous skeletal elements, the development of chondrocytes, and the mineralization and maturation of bone [13], whereas creatinine (an endogenous product of muscle metabolism whose rate of production varies depending on the muscle mass [35]) and serum calcium (representative of bone metabolism, being the skeleton an important reservoir [36]) were significantly increased in F1-MET lambs.

The lack of differences in meat chemical composition and fatty acid profile was an unexpected result, given the fact that a gene (e.g., *CPT1B*, carnitine palmitoyl transferase 1B) essential for transporting long-chain fatty acyl-CoAs from the cytoplasm into the mitochondria (where the beta-oxidation pathway takes place) was hypermethylated in F0-MET ewes, thus increasing long-chain FA in milk [13]. As stated before, the sex-dependent effect observed by Rosa-Velazquez et al. [6] in the offspring (F1) when methionine (0.1% of rumen-protected methionine) was supplied to pregnant ewes (F0) might be behind the lack of effects observed in the lipid metabolism of F1-MET male fattening lambs. These sex-dependent differences might be due to the fact that, at the same carcass weight, male lambs have more muscle per kilogram than female lambs, whereas the last ones are more prone to accumulate fat [37]. In any case, the lack of differences in the fatty acid profile is coherent with the similar lipid oxidation stability and volatile compounds observed for both groups of F1 male lambs. Oxidative stability of meat is a relevant quality trait related to the shelf life of meat in aerobic storage and consumer health, since lipid oxidation in meat leads to rancid flavor and the formation of toxic by-products [38]. One of the many factors influencing the oxidative stability of meat during storage is its fatty acid composition, with polyunsaturated fatty acids being more susceptible to oxidation [39]. The absence of differences in fatty acid profile between the meat of both groups of F1 male lambs was consistent with their similar rates of lipid oxidation, as measured by TBARS and lipid-derived volatile compounds.

Regarding color parameters, the results observed also suggest a higher oxymyoglobin content in F1-MET samples, a higher redness, and hence a more appealing color for the consumers [40]. In addition, the higher redness of F1-MET samples might be related to increased proportions of red oxidative (slow or fast) fibers in the muscle [41], and hence to differences in meat tenderness, as will be explained below. However, shear force was significantly increased (and hence, tenderness reduced) by dietary treatment, being almost 10 N higher in the F1-MET meat when compared to the F1-CTRL group, even after wet aging. A difference of 10 N is considered to be the sensory detection threshold in the degree of toughness of the meat, and by more than 40–50 N shearing force lamb meat could present problems of toughness [34,41], so consumers would be less satisfied with F1-MET meat regardless of the aging time. In this sense, Amorín et al. [42] found that methionine supplementation during cattle gestation (F0) altered the DNA methylation of the fetus, so genes of the loin related to the skeletal muscle development (e.g., myogenesis) of the calves (F1) and its physiology (e.g., mitochondrial function, among others) were differentially methylated. Although these authors did not report meat quality traits for F1 calves, those persistent differences might have affected meat quality [42] given the fact that mitochondria have an impact on oxygen consumption, energy metabolism, and apoptotic processes, which in turn affects myoglobin levels, color, and meat tenderness [43]. In agreement to this statement, it must be noted that F0-MET ewe lambs presented differential methylation of *ATP6AP1* (ATPase H+ Transporting Accessory Protein 1), a gene involved in mitochondrial pathways and also in bone development [13]. Additionally, this gene has been identified as differentially expressed in enriched pathways related to the lysosome activity, which is related to meat tenderization [44,45]. Moreover, *RLFNB* (Regulator of filamin protein B, RefilinB), a gene involved in actin filament bundle organization, was differentially methylated in F0-MET dams [13]. Therefore, intergenerational transmission of epigenetics marks from F0 to F1 (via germline) might have modified either tenderization during the aging process or the type of fiber in the muscles, thus reducing tenderness of the meat obtained from F1-MET male lambs [46]. It must be stressed that DNA methylation of male fattening lambs (F1) might have been more deeply impacted than in F0 due to the effects of methionine supplementation on the germline during the postnatal oogenesis of F0 [2]. Therefore, differential methylation in F1 of genes involved in fiber type distribution or meat tenderness (e.g., calpains, proteases, reduced sarcomere length during muscle-to-meat conversion, collagen amount and characteristics) cannot be discarded [47]. Clarifying the mechanism involved in meat tenderness using further approaches (e.g., proteomics, transcriptomics of the muscle) warrants future investigation.

## 5. Conclusions

Under the conditions of the present study, it can be concluded that 0.1% DL-methionine included in the milk replacer (DM basis) during the early post-natal life of ewe lambs (F0) does not affect feed efficiency of the male offspring (F1, male fattening lambs) neither in chemical composition nor fatty acid profile of the meat produced. However, it provokes permanent changes in the offspring (F1, male fattening lambs), thus increasing the redness, but also the hardness of the meat produced by these animals. These permanent and intergenerational effects on F1 male fattening lambs which are driven mainly by changes in the germline of F0 during postnatal oogenesis, raise concerns about the convenience of supplying methionine during the early life of animals (e.g., ewe lambs, F0) kept in the herd for breeding and replacement purposes.

## Figures and Tables

**Table 1:** Ingredients and chemical composition (g/kg dry matter unless otherwise stated) of the complete pelleted diet (CPD) administered during the fattening period of Assaf lambs.

**Ingredients, g/kg**	
Barley straw	150
Barley	413
Corn	150
Soybean meal 44	237
Molasses	20
Sodium bicarbonate	7
Ammonium chloride	2
Vitamin–mineral supplements	21
**Chemical composition, g/kg DM**	
DM, g/kg	902
^1^ aNDF	214
ADF	107
CP	191
Fat	34.5
Ash	82.1
**Fatty acid (FA) profile of the CPD, % of total FAME** ^2^	
C16:0	35
C18:2	33
C18:1	23
C18:0	5
C18:3	2.5
Others	1.5
^3^ Metabolizable energy, kcal/kg DM	2816
^3^ Metabolizable protein, g/kg DM	137

^1^ amylase-treated neutral detergent fiber.^2^ FAME: fatty acid methyl esters. ^3^ Metabolizable energy and protein was estimated according to AFRC (1993) [15].

**Table 2:** Animal performance of Assaf lambs (F1) born from ewes (F0) that were supplemented (MET) or not (CTRL) with methionine during early life.

	F1-CTRL	F1-MET	SEM	*p*-Value
Suckling period				
Live body weight at birth (kg)	5.32	4.86	0.356	0.376
Average daily gain (g/day)	214	227	23.067	0.698
Age at weaning (days)	47.8	45.3	1.709	0.306
Live body weight at weaning (kg)	15.7	15.4	0.806	0.759
Fattening period				
Dry matter intake (g/day)	1001	938	46.416	0.348
Average daily gain (g/day)	355	322	18.300	0.225
Feed conversion rate (g DMI/g ADG)	2.83	2.96	0.123	0.451
Residual feed intake (g)	9.87	−9.84	33.092	0.679
Age at slaughter (days)	87.2	88.7	0.524	0.069
Slaughter weight (kg)	29.2	29.1	1.396	0.936

SEM: standard error of the mean.

**Table 3:** Biochemical parameters of Assaf lambs (F1) born from ewes (F0) that were supplemented (MET) or not (CTRL) with methionine during early life.

	Group		Growth Stage					*p*-Value		
	F1-CTRL	F1-MET	T1	T2	T3	SEM_1_	SEM_2_	Group	Day	G*Day
Protein (g/L)	57.3	56.8	54.0 ^a^	58.4 ^b^	58.7 ^b^	0.624	0.765	0.572	0.000	0.444
Albumin (g/L)	37.1	36.9	34.2 ^a^	38.0 ^b^	38.7 ^b^	0.395	0.484	0.841	0.000	0.576
Urea (mg/dL)	35.7	34.3	25.5 ^a^	36.6 ^b^	42.9 ^c^	2.383	1.526	0.685	0.000	0.141
AST (U/L)	91.4	97.3	81.7 ^a^	96.9 ^b^	104 ^b^	3.240	3.970	0.212	0.001	0.205
GGT (U/L)	74.9	81.7	86.3 ^b^	77.9 ^ab^	70.8 ^a^	3.425	4.195	0.180	0.045	0.394
Creatine kinase (U/L)	218	337	390	212	230	60.32	73.87	0.183	0.189	0.194
Creatinine (mg/dL)	0.526	0.567	0.543	0.565	0.532	0.012	0.015	0.034	0.302	0.416
Total bilirubin(mg/L)	0.168	0.170	0.204 ^c^	0.168 ^b^	0.137 ^a^	0.006	0.008	0.874	0.000	0.713
Cholesterol (mg/dL)	56.4	58.5	75.0 ^b^	46.8 ^a^	50.5 ^b^	2.600	3.184	0.567	0.000	0.018
HDL (mg/dL)	40.6	40.4	54.3 ^b^	33.1 ^a^	34.1 ^a^	1.840	1.521	0.933	0.000	0.007
LDL (mg/dL)	14.3	14.9	16.4	13.1	14.4	1.132	1.386	0.718	0.249	0.130
Triglycerides (mg/dL)	27.2	32.5	48.1	24.5	17.1	3.566	4.367	0.310	0.000	0.184
BHB (mmol/L)	0.338	0.336	0.194	0.373	0.444	0.0559	0.0475	0.985	0.002	0.166
NEFA (mmol/L)	0.154	0.234	0.279	0.103	0.199	0.060	0.073	0.357	0.253	0.297
Glucose (mg/dL)	91.9	91.0	94.7	90.5	89.1	2.096	2.567	0.759	0.293	0.769
Insulin (uUI/mL)	31.4	31.9	13.8	24.9	52.5	11.39	13.71	0.979	0.104	0.949
Mg (mg/dL)	2.88	2.81	2.78	2.90	2.87	0.056	0.069	0.336	0.453	0.394
Ca (mg/dL)	11.2	11.6	11.3	11.6	11.4	0.089	0.109	0.016	0.130	0.110
Zn (ug/dL)	211	206	214	206	205	5.304	4.299	0.507	0.337	0.950

T1: end of suckling period; T2: mid-fattening period; T3: end of fattening period; AST: aspartate aminotransferase; GGT: gamma-glutamyl transpeptidase; HDL: high-density lipoprotein; LDL: low-density lipoprotein; BHB: beta-hydroxybutyrate; NEFA: non-esterified fatty acids; SEM_1_: standard error of the mean to compare experimental groups; SEM_2_: standard error of the mean to compare days; ^a,b,c^ means with different superscript are significantly different (*p* < 0.05).

**Table 4:** Ruminal fermentation parameters, carcass, and non-carcass traits of Assaf lambs (F1) born from ewes (F0) that were supplemented (MET) or not (CTRL) with methionine during early life.

	F1-CTRL	F1-MET	SEM	*p*-Value
Ruminal pH and fermentation end-products				
pH	5.72	5.76	0.235	0.919
Total volatile fatty acids (VFA, mmol/L)	172	186	21.061	0.632
Molar proportions (mmol/100 mmol VFA)				
Acetate	54.5	54.4	2.060	0.962
Propionate	29.6	28.8	3.832	0.892
Butyrate	10.4	11.3	1.507	0.701
Valerate	2.45	2.77	0.497	0.654
Caproate	1.04	1.11	0.366	0.889
Isobutyrate	0.7	0.71	0.243	0.981
Isovalerate	1.3	0.96	0.405	0.553
pH (LT muscle)				
0 h	6.62	6.51	0.160	0.616
45 min	6.44	6.45	0.157	0.945
24 h	5.84	5.54	0.250	0.407
Color parameters of subcutaneous fat				
L	66.3	65.6	1.439	0.736
a*	1.68	2.60	0.690	0.360
b*	13.7	14.6	1.381	0.651
Hot carcass weight (kg)	13.2	13.4	0.691	0.884
Cold carcass weight (CCW, kg)	12.7	12.9	0.668	0.875
Carcass yield (%)	43.6	44.3	0.500	0.359
Carcass components (g/kg left CCW)				
Shoulder	195	192	3.080	0.411
Loin	185	188	5.895	0.554
Rack	75	79	4.127	0.720
Neck	77	76	3.509	0.920
Belly and flank	98	98	3.276	0.704
Leg	356	354	4.486	0.798
Tail	12	13	0.735	0.758
Non-carcass components (g)				
Blood	1260	1238	89.860	0.863
Heart	175	180	18.367	0.860
Respiratory tract; pharynx, trachea, lungs	754	763	30.647	0.827
Liver	763	737	35.300	0.609
Spleen	59	67	4.003	0.155
Rumen	813	823	55.067	0.899
Fat depots (g)				
Omental fat	230	217	28.733	0.748
Mesenteric fat	277	224	30.500	0.229
Kidney knob and channel fat	109	108	13.673	0.967

SEM: standard error of the mean.

**Table 5:** Proximal composition (g/100 g raw meat) and fatty acid profile (mg/100 g raw meat) of *longissimus thoracis* corresponding to Assaf lambs (F1) born from ewes (F0) that were supplemented (MET) or not (CTRL) with methionine during early life.

	F1-CTRL	F1-MET	SEM	*p*-Value
Moisture	76.2	77.0	0.862	0.507
Protein	21.1	20.2	0.796	0.416
Fat	1.42	1.48	0.127	0.757
Ash	1.11	1.09	0.036	0.627
Total SFA	514	550	49.587	0.625
C10:0	1.48	1.70	0.253	0.565
C12:0	7.20	7.29	1.026	0.951
C14:0	24.8	34.7	5.216	0.212
C15:0 *iso*	0.26	0.25	0.064	0.925
C15:0 *anteiso*	1.55	0.72	0.367	0.142
C15:0	2.48	3.16	0.386	0.581
C16:0 *iso*	0.78	1.08	0.245	0.414
C16:0	260	291	29.62	0.488
C17:0 *anteiso*	1.92	2.08	0.590	0.863
C17:0	13.7	15.3	2.945	0.712
C18:0 *iso*	0.26	0.36	0.180	0.730
C18:0	190	184	15.203	0.802
C20:0	2.67	2.42	0.233	0.464
C22:0	4.24	4.11	0.244	0.715
C23:0	1.65	1.32	0.254	0.381
Total MUFA	507	583	56.531	0.374
C14:1c9	0.38	0.92	0.295	0.225
C16:1t9	2.23	2.84	0.594	0.495
C16:1c9	20.0	26.3	3.323	0.209
C17:1c9	5.27	6.44	1.368	0.567
C18:1t10 + t11	44.3	43.9	7.655	0.974
C18:1c9	396	460	48.573	0.383
C18:1c11 + c12	38.7	42.3	4.321	0.579
Total PUFA	241	222	18.1	0.487
C18:2t9, c12	0.85	0.93	0.565	0.926
C18:2n6	150	133	15.300	0.459
C18:3n3	4.66	4.50	0.376	0.771
C18:2c9, t11(CLA)	3.56	4.63	0.483	0.140
C18:2t10, c12(CLA)	0.86	0.35	0.289	0.244
C20:2n6	2.00	2.13	0.175	0.626
C20:3n3	1.36	1.01	0.172	0.429
C20:3n6	4.80	4.41	0.328	0.429
C20:4n6	56.3	54.5	3.802	0.759
C20:5n3 (EPA)	1.58	1.42	0.150	0.458
C22:4n6	7.77	7.64	0.521	0.857
C22:4n3	2.10	2.42	0.160	0.179
C22:5n3 (DPA)	4.72	4.33	0.305	0.391
C22:6n3 (DHA)	0.63	0.60	0.076	0.782
Total BCFA	4.88	4.65	0.882	0.861
Total OCFA	27.2	29.3	4.641	0.763
Total trans-FA	51.4	52.7	8.350	0.922
Total *n*3	14.9	14.3	0.866	0.616
Total *n*6	221	202	17.083	0.457
PUFA/SFA	0.488	0.425	0.039	0.276
*n*6/*n*3	14.9	14.3	1.041	0.688

SEM: standard error of the mean; SFA: saturated fatty acids: MUFA: monounsaturated fatty acids; PUFA: polyunsaturated fatty acids; CLA: conjugated linoleic acid; EPA: eicosapentaenoic acid; DPA: docosapentaenoic acid; DHA: docosahexaenoic acid; total BCFA: total branched chain fatty acids (C14:0 *iso*, C14:0 *anteiso*, C15:0 *iso*, C15:0 *anteiso*, C16:0 *iso*, C17:0 *iso*, C17:0 *anteiso*, C18:0 *iso*); total OCFA: total odd-chain fatty acids (C15:0, C17:0, C17:1*c*9, C23:0).

**Table 6:** Lipid stability (mg of malondialdehyde/kg of meat) of raw and cooked *longissimus lumborum* muscle corresponding to Assaf lambs (F1) born from ewes (F0) that were supplemented (MET) or not (CTRL) with methionine during early life.

	Dietary Treatments (D)			Storage Day (S)				*p*-Value		
	F1-CTRL	F1-MET	SEM_1_	0	3	7	SEM_2_	D	S	D × S
Raw meat	0.76	0.62	0.091	0.06 ^a^	0.65 ^b^	1.35 ^c^	0.112	0.307	<0.001	0.664
Cooked meat	1.82	1.46	0.172	0.184 ^a^	3.09 ^b^	-	0.172	0.143	<0.001	0.202

SEM_1_: standard error of the mean to compare dietary treatments; SEM_2_: standard error of the mean to compare storage days; ^a,b,c^: superscripts mean significant differences (*p* < 0.05).

**Table 7:** Volatile compounds (expressed in area units × 10^6^) in cooked *longissimus lumborum* muscle of Assaf lambs (F1) born from ewes (F0) that were supplemented (MET) or not (CTRL) with methionine during early life.

	Dietary Treatment (D)			Storage Day (S)			*p-*Value		
	F1-CTRL	F1-MET	SEM_1_	0	2	SEM_2_	D	S	D × S
Sum acetic acid	208	193	63.598	64.7	334	63.598	0.873	0.005	0.818
Acetic acid	41.9	43.0	9.580	44.8	40.1	9.580	0.938	0.729	0.164
Hexanoic acid	166	150	60.088	19.9	297	60.088	0.856	0.003	0.640
Sum of acyclic aldehydes	56,779	56,205	3243.192	45,694	67,290	3243.192	0.901	0.001	0.860
Pentanal	1714	1999	277.891	1678	2035	277.891	0.472	0.370	0.756
Hexanal	51,681	51,378	3032.499	41,510	61,550	3032.499	0.944	0.001	0.958
Heptanal	1515	1349	152.895	1154	1709	152.895	0.589	0.078	0.266
2-Heptenal	15.2	16.3	2.270	12.7	18.8	2.270	0.742	0.067	0.110
Octanal	780	693	125.196	608.3	864.6	125.196	0.624	0.156	0.438
2-Octenal	14.0	14.5	1.980	11.1	17.4	1.980	0.871	0.030	0.327
Nonanal	1054	750	169.694	715	1088	169.694	0.213	0.129	0.376
2-Nonenal	5.63	5.84	0.565	4.53	6.94	0.565	0.798	0.006	0.343
Decanal	9.37	8.46	1078.964	7.09	10.8	1078.964	0.555	0.022	0.393
Sum of alcohols	1953	1853	213.293	1641	2165	213.293	0.742	0.091	0.345
Butanol	17.3	10.7	3.630	8.41	19.6	3.630	0.212	0.037	0.316
1-Penten-3-ol	31.7	27.3	5.520	16.5	42.5	5.520	0.578	0.002	0.984
1-Pentanol	762	828	127.696	710	880.2	127.696	0.719	0.350	0.861
1-Hexanol	274	174	60.348	205	243	60.348	0.248	0.653	0.256
1-Heptanol	72.5	60.0	13.770	59.7	72.8	13.770	0.523	0.504	0.163
1-Octen-3-ol	605	597	112.996	527	674	112.996	0.962	0.363	0.447
2-Ethyl-1-hexanol	36.6	35.2	13.180	25.5	46.3	13.180	0.939	0.270	0.718
Methylcyclohexanol	8.73	7.63	1680.944	2.20	14.2	1680.944	0.644	0.001	0.430
2-Octenol	47.7	42.3	6.480	27.2	62.8	6.480	0.560	0.001	0.120
Octanol	88.5	62.4	17.179	52.9	98.0	17.179	0.288	0.071	0.117
Nonenol	9.19	9.17	1898.937	7.20	11.2	1898.937	0.993	0.149	0.190
Benzaldehyde	11.1	11.8	1743.942	9.90	13.0	1743.942	0.794	0.219	0.457
2-Pentylfuran	387	347	56.598	322	412	56.598	0.619	0.267	0.326
Sum of hydrocarbons	496	407	216.093	541	363	216.093	0.772	0.564	0.694
Hexane	354	240	183.894	422	171	183.894	0.661	0.339	0.518
1-Heptene	51.4	58.1	8.60	40.2	69.2	8.60	0.695	0.099	0.908
Octane	91.1	110	39.999	78.2	123	39.999	0.739	0.434	0.378
Sum of ketones	1239	1066	154.095	830	1474	154.095	0.432	0.001	0.251
2-Heptanone	122	121	14.160	93.2	150	14.160	0.986	0.001	0.173
2,3-Octanedione	1077	909	134.696	708	1279	134.696	0.385	0.006	0.281
2-Octanone	12.1	12.6	2.800	10.8	13.9	2.800	0.915	0.448	0.460
4-Nonanone	28.4	22.9	5.970	18.9	32.4	5.970	0.519	0.118	0.093
Total	61,064	60,089	3358.788	49,092	72,061	3358.788	0.838	0.001	0.778

SEM_1_: standard error of the mean to compare dietary treatments; SEM_2_: standard error of the mean to compare storage days.

**Table 8:** Color stability of *longissimus lumborum* muscle slices of Assaf lambs (F1) born from ewes (F0) that were supplemented (MET) or not with methionine (CTRL) during early life.

	Dietary Treatment (D)			Aging Days (A)			Storage Day (S)				*p-*Value					
	F1-CTRL	F1-MET	SEM_1_	0	7	SEM_2_	0	3	7	SEM_3_	D	A	D × A	S	D × S	A × S
L	41.20	41.26	0.178	41.1	41.4	0.178	40.8 ^a^	41.2 ^ab^	41.7 ^b^	0.218	0.831	0.310	0.231	0.035	0.682	0.216
a*	10.35	10.92	0.255	9.32	12.0	0.255	10.3 ^a^	11.4 ^b^	10.2 ^a^	0.221	0.134	0.001	0.640	0.018	0.887	0.325
b*	15.26	15.62	0.166	14.3	16.6	0.166	14.9 ^a^	15.9 ^b^	15.8 ^b^	0.204	0.148	0.001	0.353	0.007	0.820	0.001
a/b	0.65	0.72	0.013	0.69	0.68	0.013	0.70	0.69	0.66	0.017	0.003	0.435	0.312	0.257	0.977	0.377
R572/525	0.97	1.01	0.013	0.95	1.03	0.013	0.98	0.99	1.00	0.016	0.078	0.001	0.039	0.594	0.862	0.832
R610/525	2.21	2.79	0.042	2.56	2.43	0.042	2.52	2.51	2.46	0.052	0.001	0.036	0.074	0.701	0.654	0.612
R630/580	2.06	2.10	0.059	2.12	2.04	0.059	2.45 ^c^	2.14 ^b^	1.65 ^a^	0.072	0.632	0.363	0.937	0.001	0.946	0.087

SEM_1_: standard error of the mean to compare dietary treatments; SEM_2_: standard error of the mean to compare aging days; SEM_3_: standard error of the mean to compare storage days; ^a,b,c^: superscripts mean significant differences, Tukey test (*p* < 0.05); R630/R580: reflectance ratio at 610 nm/525 nm; R610/R525: reflectance ratio at 630 nm/580 nm (AMSA, 2012) [27]; D × S × A interaction was not significant for any of the parameters included in Table 8.

**Table 9:** Changes in cooking losses (CL, %) and shear force (SF, N) of *longissimus lumborum* muscle of Assaf lambs (F1) born from ewes (F0) that were supplemented (MET) or not with methionine (CTRL) during early life.

	Dietary Treatment (D)			Aging Days (A)				*p-*Value		
	F1-CTRL	F1-MET	SEM_1_	0	3	7	SEM_2_	D	A	D × A
Cooking losses	15.2	15.5	0.336	13.8	15.7	16.6	0.421	0.576	<0.001	0.082
Shear force	56.0 ^a^	64.6 ^b^	2.137	66.9 ^b^	61.2 ^b^	52.8 ^a^	2.674	0.007	0.002	0.841

SEM_1_: standard error of the mean to compare dietary treatments; SEM_2_: standard error of the mean to compare aging days. ^a,b^: superscripts mean significant differences (*p* < 0.05).

## Data Availability

Data will be made available under request.

## Supplementary Materials

The following supporting information can be downloaded at: https://www.mdpi.com/article/10.3390/ani15091290/s1, Figure S1: Portions or slices obtained from the *longissimus lumborum* muscles (right side or left side, randomly chosen) and analysis carried out with each portion; Table S1: Analyses carried out in *longissimus lumborum* muscle and treatments of meat before analysis.
