# Supplementary material for: The Early Methionine Supplementation of Ewe Lambs (F0) Modifies Meat Quality Traits of the Progeny (F1, Male Fattening Lambs)
Source: Animals (Basel). 2025 Apr 30;15(9):1290. doi: 10.3390/ani15091290 (PMC12071116; doi:10.3390/ani15091290)
Supplement: Supplementary file 1 [file animals-15-01290-s001.zip › animals-3579777-supplementary figure.pdf]

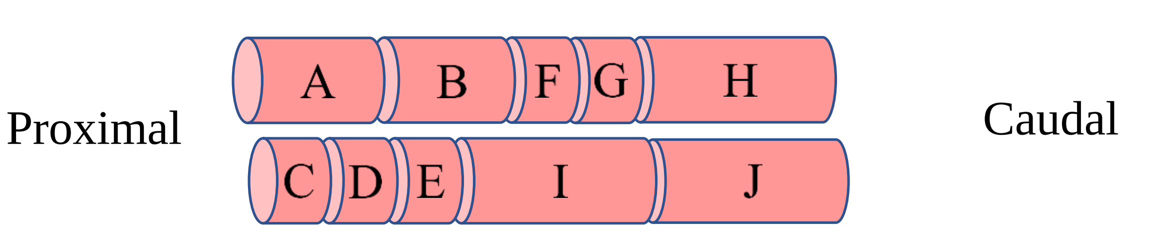

A and B: Colour stability in non-aged and 7-day-aged raw meat, respectively.
C, D and E: Lipid oxidation in non-aged raw meat on days 0, 3 and 7, respectively, of refrigerated aerobic storage.
F and G: Lipid oxidation and volatile compounds in cooked (non-aged) meat just after cooking and after 2-day-storage period, respectively.
H, I and J: Cooking losses and shear force in non-aged, 3-day- and 7-day-aged raw meat after cooking.

Figure S1. Portions or slices obtained from the *longissimus lumborum* muscles (right side or left side, randomly chosen) and analysis carried out with each portion.
