# Supplementary material for: The Early Methionine Supplementation of Ewe Lambs (F0) Modifies Meat Quality Traits of the Progeny (F1, Male Fattening Lambs)
Source: Animals (Basel). 2025 Apr 30;15(9):1290. doi: 10.3390/ani15091290 (PMC12071116; doi:10.3390/ani15091290)
Supplement: Supplementary file 1 [file animals-15-01290-s001.zip › animals-3579777-supplementary table.pdf]

Table S1. Analyses carried out in *longissimus lumborum* muscle and treatments of meat before analysis

	Wet ageing	Cooking	Aerobic refrigerated storage
Lipid oxidative stability	No	Raw	0, 3 and 7 days
		Cooked	0 and 2 days
Volatile compound changes	No	Cooked	0 and 2 days
Colour stability	0 and 7 days	Raw	0, 3 and 7 days
Cooking losses and texture	0, 3 and 7 days	Cooked	No
